# Editorial: The impact of internal and external influences on memory and their relevance to legal decisions

**DOI:** 10.3389/fpsyg.2024.1408797

**Published:** 2024-05-29

**Authors:** Fabiana Battista, Ivan Mangiulli, Henry Otgaar, Antonietta Curci

**Affiliations:** ^1^Department of Education, Psychology, Communication, University of Bari Aldo Moro, Bari, Italy; ^2^Faculty of Law and Criminology, Leuven Institute of Criminology, Katholieke Universiteit (KU) Leuven, Leuven, Belgium; ^3^Faculty of Psychology and Neuroscience, Maastricht University, Maastricht, Netherlands

**Keywords:** internal influences, external influences, forgetting, amnesia, memory distortions, false memories

## Introduction

Cases of wrongful convictions based on unreliable testimonies, as shown by data from Innocence Projects of different countries (i.e., projects aiming to assure a fair process to people wrongfully convicted), show the deleterious effects of inaccurate memories in the legal context. Both external and internal influences can make memories inaccurate. For example, an abundance of research has shown that exposure to misleading and suggestive information can undermine memory for the original event even resulting into formation of false memories (for a review, Pickrell et al., 2016). Similarly, studies on deception have also found undermining effects in terms of both forgetting and false memories for the event (for a review, Battista and Otgaar, 2022). In the current Research Topic, we provide a unique assemblage of empirical and theoretical papers on these different influences on memory and the impact of memory studies in the courtroom. Specifically, in this Research Topic, papers on emotions and memory, traumatic memories, memory conformity, the misinformation effect, lying and memory, and developmental trends in false memories are presented.

## Articles on external influences

Specifically, Marr et al. wrote a critical view on how acute stress can influence the retrieval of events. Their article is a reply to Pezdek and Reisberg's (2022) manuscript on whether or not stress can improve memory retrieval. The authors reviewed the literature on acute stress and memory, concluding that evidence on the link between them is mixed thereby arguing that the relationship between stress and memory depends on several moderators.

Davis et al. also wrote a review on the role of stress on memory but accounting for how stress affects attention which, in turn, can impact the encoding and retrieval of emotional events. In their review, they challenged common claims regarding tunnel memory, the effects of attention narrowing under stress, and the accuracy of memory for emotionally intense events, discussing potential biases in the acceptance of these claims by legal professionals. Finally, they reflected on the role of emotion in the interpretation and memory of sexual consent as well as the potential of trauma-informed interviewing strategies to induce memory distortions.

Dodier et al. provided a new framework to understand the phenomenon of recovered memories. They proposed that recovered memories can be seen as a form of involuntary autobiographical memories whose retrieval is triggered by internal (e.g., age and internal cues) or external cues (e.g., suggestion in therapy, suggestion during interview, and contextual cue). Using this framework, they proposed a new way to evaluate the validity of recovered memories in legal context and provided guidelines for practitioners to correctly apply this novel approach.

Kękuś et al. demonstrated that the classical memory conformity effect occurs also in online situations (i.e., MORI-v) and the effect is comparable to the effects obtained from in-person studies. They also showed the role of individual traits, such as susceptibility to social influence, need for closure, and self-esteem, on the memory conformity effect.

In two studies, Cullen et al. examined the influence of different types of misinformation (i.e., pro-prosecution, pro-defense, or contradictory) on juror decision-making and memory. Specifically, they tested the effects of congruent misinformation on jurors' evaluation of the credibility and verdict for a fictitious trial record of an alleged sexual assault as well as for the recall of the case.

Shah and Knott presented an experiment aiming to test the influence of retention interval and arousal for negative events on the exposure to gist or verbatim misleading information. They demonstrated that the misinformation effect is strong enough to persist over time for negative highly arousing event. According to the authors, these results further suggest the urge to avoid suggestive interviews, especially when arousing events are at stake.

O'Donnell et al. explored two possible aspects affecting the effects of misinformation on memory: The misinformation repetition and the source of misinformation. In two experiments, they readapted Foster et al.'s (2012) procedure and consistently detected in both studies that repetition did affect people's proneness to report misinformation in their recall for the original event, while source of misinformation did not.

Deering et al. further tested the misinformation effect by combining the misinformation procedure with a procedure used in line-up identification studies. They demonstrated that the viewing angle congruency between the perpetrator seen in the encoding phase and the one seen in the misinformation phase did not affect the identification accuracy. The authors concluded that the congruency between encoded and misleading information does not determine either an increase or decrease in the misinformation effect.

Jones et al. extended research on the identification of a culprit in line-up situations by taking into account the phenotypic bias (e.g., tendency to associate people with more Afrocentric -as opposed to Eurocentric- features with criminality). In their study, phenotypic bias did not undermine the correct recognition of the culprit when the culprit had more Afrocentric, rather than Eurocentric, features. Instead, participants were more able to identify the culprit when the phenotype was incongruent between the culprit and the line-up fillers, suggesting that practitioners (i.e., police) need to keep in mind the importance of matching facial phenotype between suspects and fillers when they arrange line-ups.

## Articles on internal influences

Dianiska and Meissner investigated the effect of lying on memory accuracy and consistency. In addition, they examined whether the type of interview (i.e., Structure Interview, Reverse Order Interview) influences these two memory outcomes. Overall, lying made people's recall of the original event less accurate along with making people less consistent across interviews. Moreover, interviewing people with a Reverse Order technique reduced inconsistencies, in terms of omissions.

## Articles on internal and external influences

Rosendaul et al. summarized studies on the two lines of research of the normative developmental position and the reverse developmental position. By reviewing internal (e.g., source misattributions, inferential reasoning) and external (e.g., valence, suggestion) influences that affect people's proneness to false memories, the authors argued there are no conclusive findings on how age determines memory accuracy, as such both children and adults can be reliable sources of information during legal proceedings.

Relatedly, Otgaar et al. provided an overview of studies on the role of suggesting non-occurrence and non-experience (i.e., external influences) and on the effect of deception (i.e., internal influence) on forgetting and false memories. According to this research, the authors map the outcomes associated to both influences underlining that, although differently, both types of influences can lead to similar mnemonic effects. Cognitive dissonance is put forward as the mechanism behind and operating both at an interpersonal or intrapersonal level.

## Conclusions

The articles collected in this Research Topic show the wide researchers' interest in both internal and external influences, with a higher inclination to study external influences than internal ones. This could be due to several reasons. For one thing, while it is easier to explore external influences compared to internal ones, there is a clear necessity for fresh insights in these areas. Hence, there is a call to replicate previous findings in different contexts or through methodological adaptations to uncover novel insights. In addition, the few studies on internal influences and memory invite future investigation in this regard.

To conclude, the studies in this Research Topic provide legal practitioners with practical information on how to avoid detrimental effects on memory and legal decisions. We firmly think that the current Research Topic can inspire future studies, contributing to disseminate knowledge among legal professionals.

## Author contributions

FB: Conceptualization, Writing – original draft, Writing – review & editing. IM: Writing – review & editing. HO: Writing – review & editing. AC: Writing – review & editing.
